# Near-Infrared-Responsive Cancer Photothermal and Photodynamic Therapy Using Gold Nanoparticles

**DOI:** 10.3390/polym10090961

**Published:** 2018-08-30

**Authors:** Hyung Shik Kim, Dong Yun Lee

**Affiliations:** 1 Departments of Bioengineering, College of Engineering, Hanyang University, Seoul 04763, Korea; kimhs5774@naver.com; 2 BK21 PLUS Future Biopharmaceutical Human Resources Training and Research Team, and Institute of Nano Science & Technology (INST), Seoul 04763, Korea

**Keywords:** gold nanoparticle, photo thermal therapy (PTT), photo dynamic therapy (PDT), photosensitizer (PS), near infrared resonance (NIR)

## Abstract

Rapid growth of nanotechnology is one of the most quickly emerging tendencies in cancer therapy. Gold nanoparticles roused a distinctive interest in the field, due to their incomparable light-to-thermal energy conversion efficiency, and their ability to load and deliver a variety of anticancer drugs. Therefore, simultaneous photothermal (PTT) and photodynamic (PDT) cancer therapy is available by the role of the thermal agent of the gold nanoparticle itself and the drug delivery carrier for photosensitizer (PS) transport. In this review, the physical, chemical, and biological properties of gold nanoparticle, which can promote PTT and PDT efficiency, are briefly demonstrated, and we highlight recent progression in the development of PS-containing gold nanocomposites for effective cancer therapy.

## 1. Introduction

**Figure 1:**
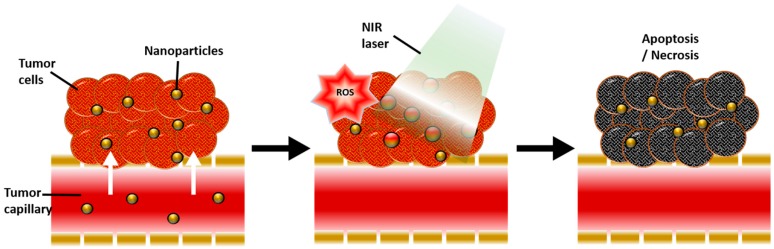
Schematic illustration of the physiological and biological effects of gold nanoparticle-mediated photothermal therapy (PTT) and photodynamic therapy (PDT). A large amount of gold nanoparticles accumulate due to the leaky vasculature of the tumor, resulting in a photothermal effect in response to near-infrared (NIR) light and reactive oxygen species (ROS) generated by secondary delivered photosensitizer (PS), ultimately inducing apoptosis and necrosis of tumor tissue.

**Figure 2:**
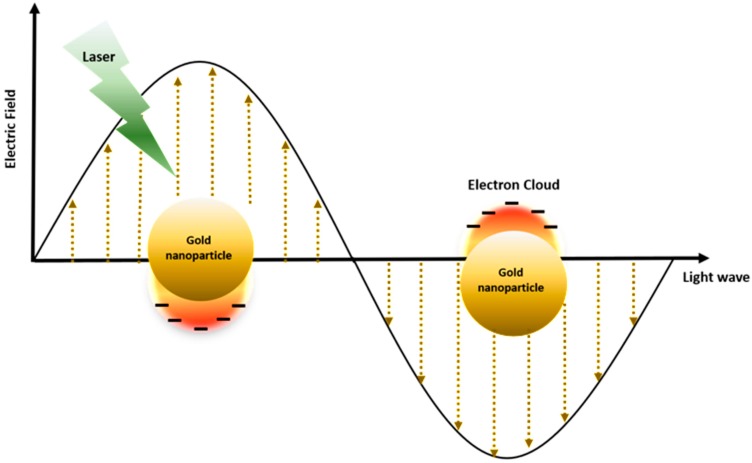
The localized surface plasmon resonance (LSPR) phenomenon on the surface of gold nanoparticles under irradiation at specific light wavelengths.

Photothermal therapy (PTT) and photodynamic therapy (PDT) are promising cancer treatments in which the heat generation of nanoparticles and the activation of photosensitizer (PS) drugs occurs in response to exogenously applied specific wavelengths of light. In the presence of nanoparticles and PS drugs under external photo-activating light, cytotoxic photothermal heating via the surface plasmon resonance (SPR) phenomenon and reactive oxygen species (ROS) can trigger apoptotic and necrotic cancer cell death [1,2] (Figure 1). Gold nanoparticles have been considered for both photothermal agents and photosensitizer carriers, due to their surface plasmon resonance (SPR) effect, which has a high efficiency of light-to-heat conversion, and simple thiolation chemistry for the functionalization of desired molecules, enhancing its capability for loading PS drugs. In the case of PTT, collective oscillation of electrons on the surface causes SPR at specific frequencies of light, resulting in strong extinction of electrons, and consequently generating hyperthermia [3] (Figure 2). In addition, certain types of tumor targeting can be enhanced with gold nanoparticles that have significant nano-scale benefits that can be easily accumulated in tumor tissue by the enhanced permeability and retention (EPR) effect of the tumor microenvironment [4]. Furthermore, due to thiol-abundant chemistry of the gold nanoparticle surface, it can be easily functionalized with various tumor targeting moieties, progressively fortify the tumor targeting aspect.

Recent studies have shown that photothermal (PTT) and photodynamic (PDT) treatments that respond to near-infrared (NIR) wavelength lasers through the combination of multifunctional plasmon nanoparticles and fluorescent photodynamic agents can achieve a synergistic effect for tumor therapy [5,6,7,8]. Most photosensitizers (PS) are hydrophobic and they require a delivery system to accomplish their tumor therapeutic effects. Much effort has been devoted to the development of dual PTT and PDT therapeutics consisting of a combination of several types of gold nanoparticles and photosensitizers to eradicate tumors. Nonetheless, additional research is needed to improve treatment protocols by regulating optical delivery, power density, and irradiation dose to establish clinical feasibility. Here, we focused on the recent progress in gold nanoparticle-mediated photothermal and photodynamic dual cancer therapies, mainly categorized by different structures of gold nanoparticles and species of photosensitizer (PS) to accomplish PTT and PDT, and lastly we comment on their academic developments and advances.

## 2. Physicochemical Properties of Gold Nanoparticles for Photothermal Therapy

**Figure 3:**
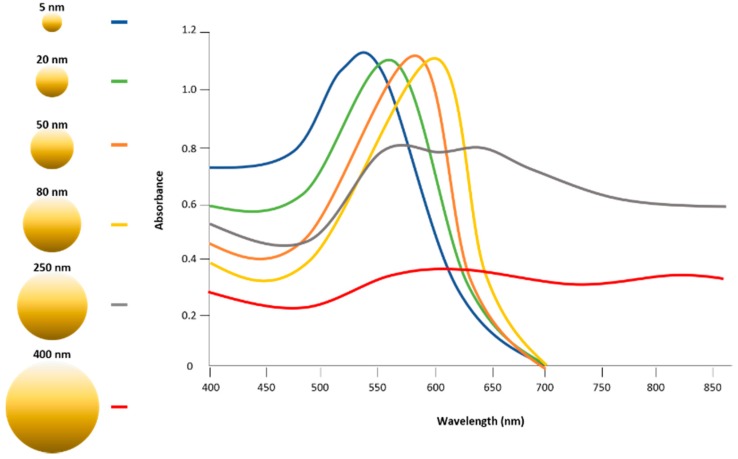
Absorption spectra of UV-visible spectrometry, depending on the diameter of gold nanoparticles, 5 to 400 nm, demonstrating a transition of specific wavelengths that generate surface plasmon resonance.

Gold nanoparticles are attracting attention as applications in cancer therapy due to the important resonance characteristics achieved by energy excitation, which are obtained by the application of light of a specific wavelength to the surface of the gold nanoparticles (Figure 3).

Under a light wavelength of a certain frequency, the collective oscillation of the surface electrons causes SPR, resulting in the strong extinction of electrons (Figure 2). Gold nanoparticles represent specific wavelengths, emission frequencies, and emission wavelengths that are highly dependent on the size, shape, surface, and aggregation state of the nanoparticles. There are various nanoparticle shapes and sizes such as nanocage, nanoflower, nanoshell, nanosphere, nanorod, nanostar, and nanoporous gold disks. Each nanoparticle contains different wavelengths representing the maximum excitation of their electrons, and exhibits a unique light-to-heat conversion efficiency from 22% to 103%, depending on size, surface, shape, and aggregation state (Table 1).

Gold nanoparticles in the 10–200 nm range can perform local oscillated local surface plasmon resonance (LSPR) with coherent free-conduction band electrons at specific wavelength illumination (Figure 2). The electrons under the “conduction band” can be strongly polarized, where the incident light resonates with the wavelength of the surface plasmon of the nanoparticles. Electrons under the influence of the external field vibrate to create a net charge difference at the nanoparticle boundary. Incident light can be absorbed and scattered simultaneously by gold nanoparticles, and “photon confinement” generates strong electromagnetic fields on the particle surface and causes various optical phenomena [50].

Recently, several kinds of gold nanoparticles have been studied. A comparison of the photothermal heating capacity of each particle type should take into account factors such as wavelength energy absorbance/scattering rate and thermal heat dissipation rate.

As a result, there are distinct advantages to these elements among different types of gold nanoparticles, depending on the shape and diameter of the gold nanoparticles.

The absorbance/scattering ratio was the largest in nanoparticles with smaller radii. Thus, nanoparticles of small volume were more efficient optical transducers. However, as smaller nanoparticles decrease extinction, their effects may be limited [51]. Experimental conversion efficiencies were higher due to scattering in the solution and subsequent reabsorption potential, which increased with nanoparticle size. For gold nanoparticles with these various dimensions, it cannot be concluded that any particular type is best suited for cancer treatment, because of their respective advantages and features. Therefore, it is very important to select gold nanoparticles with specific advantages for each purpose. Nonetheless, the observed trends are similar to experimental data and further demonstrate the size effect of photothermal heating [12].

## 3. Photothermal Therapy of Gold Nanoparticles

Recently, many researchers have investigated PTT of gold nanoparticles, both in vitro and in vivo. Gold nanoparticles are very important agents for PTT in nanomedicine. Therefore, gold nanoparticles have been used to damage cancer cells, viruses, and bacteria, based on heating effects under laser irradiation due to increased absorption induced by the distinguished properties of LSPR [32,52,53] (Figure 1). The plasmon resonance peak of GNPs can be adjusted to the near-infrared region by controlling the geometrical and physical parameters of the nanostructures, such as size and shape, which mainly contribute to the penetration depth efficacy of PTT [54,55,56] Many researchers are focusing on the photothermal therapy of gold nanoparticles of different sizes and shapes, such as gold nanocages, gold nanorods, gold spheres, gold nanoshells, gold nanoflowers, and gold nanorings [5,7,10,11,14,16,17,18,19,26,27,28,29,30,31,32,33,34,35,36,37,38,39,41,42,43,44,46,47,49].

Furthermore, there have been anti-cancer phototherapy experiments using modified gold nanoparticles such as nanorods and nanoshells. As their name suggests, these particles consist of gold surface with hollow centers, and they can be used to store enzymes or drugs, unlike simple gold nanoparticles. This fabrication was made possible by the tunable nature of the size and shape of the gold nanoparticles, which can be tuned to specific wavelengths for each purpose. Because gold nanorods have higher absorption cross-sections at near-infrared frequencies per unit, they are used more in certain applications than other types of nanoparticles, and it is easier to control the unique longitudinal micellar shape in the surfactant solution [57].

Nanorods also dramatically reduces the radiated attenuation effect, resulting in narrower linewidths compared to spherical nanoparticles at comparable resonant frequencies [58]. These advantages brought efficiency when converting light energy into heat energy. This research team designated areas of a culture dish containing KB cells (cancer cell line derived from oral epithelium) treated with folate-functionalized nanorods to directly target tumor cell membranes. The results showed extensive damage to the tumor cell membrane due to intense local heating generated by the nanorods, and a folate-functionalized target portion at a low light output density of 44 W/cm^2^ [59].

At the molecular level, hyperthermia of PTT-mediated gold nanoparticles induces cell shrinkage, protein denaturation, and membrane rupture [60]. Hyperthermia-induced cytotoxicity occurred within 1 h at 42 °C, which could be shortened to 3–4 min using higher temperatures, such as 80 °C [60,61,62,63]. This finding has shown that cytotoxicity induced by hyperthermia depends on the reaction time and the concentration of the gold nanoparticles. High-temperature-induced cytotoxicity was more pronounced under appropriate wavelengths of laser irradiation. Also, since gold nanoparticles have specific target moieties, this effect is cancer cell-specific.

Much effort has been devoted to targeting gold nanoparticles around tumor cells to increase the dosage of gold nanoparticles at sites where PTT is needed, and to minimize the side effects to the normal torso. To this end, the surface of the gold nanoparticles was transformed to a specific site that targets cancer cells. For example, to actively target Hodgkin’s cells, gold nanoparticles were chemically conjugated with BerH2 and ACT1 antibodies to specifically bind to CD30 and CD25 receptor molecules overexpressed on the L-428 Hodgkin’s cell surface [60]. These nanoparticles were easily synthesized due to their efficient bioconjugation and preservable photothermal properties, even after chemical and physical reactions. Others conjugated gold nanospheres to anti-EGFR antibodies in order to image and treat oral tumor cells in vitro using a continuous argon laser at 514 nm, the maximum absorbance of 40 nanometer particles. Cancer cells targeted with nanoparticles showed a 2- to 3-fold reduction in laser power compared to normal cancer cells [29]. Other researchers have used specific monoclonal antibodies to treat tumor cells. Ig-bound 30 nm gold nanoparticles can form 10–20 clusters on the surface of the cell membrane. K562 cells were irradiated with a single laser pulse at 5 U/cm^2^ of optical fluence at a wavelength of 532 nm, and cells lacking the specific antibody were hardly damaged, and cells targeted to specific antibodies were completely destroyed [27].

Using a silica–gold nanoshell labeled with trastuzumab or anti-HER2, the team demonstrated the usefulness of gold nanoshell in trastuzumab-resistant breast cancer and metastatic cancer. The mechanism of trastuzumab resistance on cancer has been reported in various ways, including the overexpression of insulin-like growth factor I (IGF-1) [64], overexpression of glycoprotein MUC4 [65], and constitutive P13K/Akt activity [66]. HER2 receptor mutations lead to trastuzumab resistance in breast cancer treatment, but there are extracellular components that are not mutated in the HER2 receptor. This suggested the possibility of using antibodies against the HER2 receptor as a targeting method for gold nanoshell-mediated phototherapy. Thus, research on two trastuzumab-resistant breast cancer cell lines, i.e., BT474 AZ LR and JIMT-1, was conducted. The results showed that gold nanoshell-based photothermal therapy could potentially be used in therapy-resistant breast cancer resection [67]. Furthermore, another experiment with gold nanoshells conducted on the breast carcinoma cancer cell lines [33]. Cell viability was not affected by gold nanoshell when cells were incubated with gold nanoshells in the absence of laser irradiation. Breast carcinoma cancer cells were incubated with PEG functionalized gold nanoshells, resulting in irreversible photothermal toxicity after 4 min irradiation with CW NIR light (diode laser, 820 nm, 35 W/cm^2^). Deviation of temperature was more than 30 °C resulted in tissue damage, cell shrinkage, coagulation, and the loss of nuclear staining. In contrast, there was no significant cell death from 4 min irradiation with only laser treatment (4 W/cm^2^) without nanoshell incubation [33].

Recent researchers have demonstrated the combination of photothermal therapies and diagnostic methods with in vivo imaging using multifunctional nanocomposites. Gold nanoparticle-mediated cancer therapy is a good opportunity because of the enhanced permeability and retention (EPR) effects of the tumor vasculature, and the ease of transport to the tumor tissue. These advantages allow surface-functionalized gold nanoparticles used in cancer therapy to be passively absorbed into tumor parenchyma. One team has shown a therapeutic effect on tumors treated with untreated gold nanoparticle-mediated phototherapy. The results were necrosis by day 10, and no tumor regeneration for 90 days [32].

In contrast, some researchers have focused on the microenvironment of tumor sites to optimize the therapeutic effect of specific delivery of gold nanoparticles, and the photothermal effects of particles. In recent studies, macrophages and neural stem cells (NSCs) have received great interest as tumor-targeting tumors, due to their unique tumor index properties. The tropism of stem cells and macrophages was evidenced by the expression of specific tumor secretion factors. Tumor tissues generally undergo local synthesis of anti-cancer agents such as inflammatory cytokines and chemokines. Some of these several macrophage and stem cell-attracting factors become homing effects for the cancer region [68].

Research has also been carried out to specifically target cancer sites by combining the PTT effect of gold nanoparticles with the tumor-induced effects of stem cells. In the endosomes of cells, mesenchymal stem cells (MSCs), which enable the aggregation of pH-sensitive gold nanoparticles (PS-AuNPs), can be vectors for photothermal therapy, and they can especially be used for target tumors, which reveals a mild-acidic environment. These cohesive structures enable non-exocytosis of particles, and they have a higher cellular retention compared to pH-insensitive AuNPs (cAuNPs). PS-AuNP-containing MSCs (MSC-PS-AuNPs) injected intravenously into tumor-bearing mice models exhibited a 37-fold higher tumor targeting efficiency (5.6% of the injected dose) due to the tumor-orientation of the MSCs. This prevents exocytosis of large PS-AuNP aggregates and an 8.3 °C higher heat generation compared to injections of cAuNPs after specific NIR laser irradiation, which resulted in an overwhelming anticancer effect [69]. This research showed the high tumor targeting efficiency of MSCs and large aggregation of pH-sensitive gold nanoparticles in acidic tumor tissue and endosomes.

A variety of techniques have been explored to avoid damage to normal cells by the accumulation of gold nanoparticles locally in tumorigenic areas through drug delivery methods using biological aspects. Based on further technological advances, there is a great potential to reduce side effects and efficiently target tumors.

## 4. Enhanced Photodynamic Therapy Mediated by Gold Nanoparticle

Most of the family of photosensitizers belongs to the porphyrin class, but they also include various compounds and their variations. An ideal photosensitizer should consider a number of principal requirements, stability in physiological solutions, established physical and chemical properties, minimal dark toxicity, intense absorption of red or near-infrared light that penetrates deeper into biological tissues, tumor-tissue accumulation efficiency, its interaction with the distribution dynamics, effective and long-term free radical and singlet oxygen generation, and, finally, production and commercial availability that offers the possibility of successfully introducing drugs into the clinical practice, side effects, and elimination kinetics [70]. If PS is chosen well, PDT is typically characterized by low morbidity, good tolerance, minimal invasive procedures, repeated use at the same site, minimal dysfunction, and generally outpatient treatment, and it is a promising therapy for treating cancer and other malignant diseases [71,72].

To overcome many of the drawbacks of currently used photosensitizers, many researchers are working to develop gold-containing nanocomposite photosensitizers that have unique properties that can overcome some of the limitations of PDT applications and improve efficiency. Gold nanoparticles are a good biocompatible carrier of PS, especially in hydrophobic formulations [73,74,75]. In addition, PS molecules can be encapsulated or conjugated on the surface of the gold nanoparticle. Each option has advantages and disadvantages. After reaching the tumor, the PS molecules on the NP surface can either spontaneously (non-covalently) or enzymatically (covalent attachment) separate from the nanocarrier, adhere to the prokaryotic membrane, and finally induce tumor destruction after light irradiation [6,18]. The photoactivity of PS may be interfered with by the gold nanoparticle; however, at the same time, the PS inside the NP is better protected from destruction in the environment of living organisms on the way to the tumor compared to the surface location [76].

A number of groups have reported that gold nanoparticles enhance not only the accumulation of PS, but also the development of reactive oxygen species (ROS), a fundamental property of photodynamic therapy when combining PS, such as hematoporphyrin, toluidine blue O, indocyanine green, AlPcS4, and phthalocyanine [77,78]. Of the PSs actively studied for the generation of nanocomposites, phthalocyanine occupies a prominent position due to its high extinction coefficient for far-infrared (~670 nm), the excited state of high quantum yield, and easy functionalization. The authors have demonstrated that phthalocyanine-stabilized gold nanoparticles can produce cytotoxic singlet oxygen. In this paper, gold nanoparticles was functionalized with a photosensitizer phthalocyanine (Pc), and GNP was used with the TOAB phase transfer reagent used during synthesis. They suggested that gold nanoparticles can be used to efficiently deliver photosensitizers in photodynamic therapy to improve the cytotoxicity of photosensitizers. They went through experiment that compounds (photosensitizer/gold/phase transfer reagents) have been shown to achieve higher singlet oxygen species generation (SOG) compared to free photosensitizers [79]. Subsequently, for the water-soluble and biocompatible properties, a PEGylated gold nanoparticle-silicon phthalocyanine 4 (Pc 4) complex was synthesized, which can be used to transfer hydrophobic drugs to the PDT site. The results of in vitro and in vivo drug release experiments indicate that drug delivery is highly efficient, and that passive accumulation prefers tumor sites. Compared with conventional PDT drug delivery in vivo, PEGylated gold nanoparticle increased Pc4 administration approximately 2-fold. In vivo treatment did not show any obvious side effects except that Pc4 was found in whole mouse body including lung and kidney. Within one week after PDT, tumor necrosis and tumor size were reduced because of the therapeutic effect [80].

For better tumor targeting and accumulation of PS at the region of the tumor, gold nanoparticles were complexed with peptides for both epidermal growth factor and transferrin receptors that were over-expressed on the surface of polymorphic glioblastoma. Gold nanoparticles were simultaneously used as delivery media for the therapeutic drug sensitizer phthalocyanine 4 (Pc4) [80]. As Pc4 is a promising photodynamic therapy drug that is ultimately activated by the appropriate wavelength of light to produce cytotoxic ROS, the result was that the dual targeted gold nanoparticles delivered Pc4 into tumor cells as compared to non-target cells in a manner that was five to six times more effective, and there was a significant improvement over single target gold nanoparticles [81,82]. In another study, the researchers studied the in vivo PDT efficacy of intravenously injecting C11Pc (phthalocyanine derivative)-conjugated AuNP into subcutaneously transplanted murine melanoma (B78H1 cells) in mice. Compared to the free C11Pc, the AuNP-C11Pc conjugate was found to more selectively target cancer tissues. In addition, it induced a broader PDT response by promoting an antiangiogenic response by causing extensive damage to blood capillary and endothelial cells. However, the AuNP-C11Pc conjugate was absorbed by the spleen and liver, and its persistence in the liver lasted up to one week without any apparent reduction in PS [83]. To overcome these drawbacks, they reported that PEGylated (HS–PEG–COOH) antibody was conjugated to the HER2 monoclonal antibody to target PEGylated AuNP-C11Pc conjugate to breast cancer cells. In vitro experiments demonstrated selective targeting of the 4-component “antibody-C11Pc-PEGAuNPs” conjugates to breast cancer cells overexpressing the HER2 epithelial cell growth factor receptor, and efficacy in PDT applications, but no in vivo results were presented [84].

On the other hand, some of the study groups synthesized a gold nanoparticle for PDT and hematoporphyrin conjugates, and compared the PDT efficiency of the hematoporphyrin–gold nanocomposite with different diameters in vitro. It was found that the larger the particle, the better the performance of nanostructures, with a gold nanoparticle of 45 nm compared to a 15 nm gold nanoparticle, since porphyrin molecules can be carried more efficiently into malignant cells [77,85,86]. One of the group asserted that the enhanced generation of ROS from Protoporphyrin IX by gold nanoparticles is said to be size dependent. Larger-sized gold nanoparticles have a stronger ability to increase the ROS generation of photosensitizers due to strong scattering electromagnetic (EM) fields around the particles, compared to smaller particles. However, cellular PDT efficacy was dependent not only on ROS production, but also on the size-dependent cellular uptake of AuNP.

Research shows that combining phototherapy with photodynamic therapy is very useful for treating cancer, which is thought to have better cell killing efficacy. Other groups also demonstrated the synergistic effects of PTT for PDT with different wavelengths, different species of photosensitizers, different gold nanostructures, and other cell lines [19,43,87,88]. One of them is a chlorin e6- (Ce6-), aptamer switch probe- (ASP-), gold nanorod (AuNRs) complex designed for multimodal cancer therapy. In their study, when a complex is contacted with a target cancer cell, the Ce6 molecule migrates away from the gold surface due to structural changes in the ASP and produces singlet oxygen under light irradiation to the PDT. At the same time gold nanoparticle can kill cells through the PTT method due to its high absorption efficiency. In addition, the team has developed a new multifunctional theranostic platform for cancer treatment and imaging. The monolayer of the assembled gold nanoparticles was a vesicle that could contain a Ce6 photosensitizer. Because gold vesicles have a strong absorbance at wavelengths of 650–800 nm, adjacent GNPs of the vesicle membrane can combine plasmons together. This enables excitation of gold vesicles and Ce6 with 671 nm laser irradiation to generate high heat and singlet oxygen for cancer cell killing. Both in vitro and in vivo treatment results demonstrated that the therapeutic efficacy of GV-Ce6 was improved over that of PTT/PDT alone, due to the individual PTT or PDT alone, or the coordinated effect of PTT/PDT [5]. The other groups performed PDT + PTT treatment for the first time in synergy with large solid tumors in vivo, using the GNR/SiO_2_–HP (hematoporphyrin) complex. A wide range of tumor necrosis occurred, and tumor volume decreased sharply compared with PDT plus PTT and PDT alone [21].

## 5. Conclusions and Perspectives

AuNP is an ideal light-to-heat transducer because its optical properties can be adjusted by adjusting the structural dimensions to strongly absorb tissue-penetrating NIR light. AuNP-mediated PTT has been thoroughly studied as a stand-alone cancer therapy, but its use improves with PDT and treatment methods. Applying gold nanoparticles in the field of PDT is a very promising approach for future technological advances, and gold nanoparticles have tremendous potential for tumor therapy. However there still exist limitations of recent PTT and PDT technology for clinical trials. Firstly, the quantification of nanoparticle in tumors and aspects of biodistribution in the specific organ have to be addressed. Second, the penetration depth of NIR light should be enhanced and monitored.

In summary, there has been considerable progress in the development of GNP-mediated multifunctional nanoparticle systems, and it is likely that a co-treatment strategy to improve cancer treatment efficiency will be introduced in the near future. However, these applications need to overcome critical barriers before being transferred to the clinic, such as the non-biodegradability and the low penetration depth of GNP.

## Figures and Tables

**Table 1:** The summary of photothermal conversion efficiencies of gold nanoparticles of various shapes and PTT or PDT application.

Type of Gold	Size	Photothermal Conversion Efficacy	Laser	Ref.	Treatment	Application	Brief Mechanism	Ref.
Gold nanorods	17 × 56 nm	22%	0.4 W/cm^2^, 808 nm	[9]	PTT	In vitro cell eradication	Specific targeted, NIR wavelength	[10,11]
	10 × 38 nm	95%	CW laser, 809 nm	[12]		In vivo cancer treatment	Nontargeted, NIR wavelength	[11]
	13 × 44 nm	55%	815 nm	[13]		In vivo cancer treatment	Specific targeted GNRs laden macrophages, NIR wavelength	[14]
	7 × 26 nm	50%	2 W/cm^2^, 808 nm	[15]	PDT	In vitro Cell eradication PS delivery	Single light wavelength both for PTT and PDT, Specific targeted	[16,17,18,19]
						In vitro and in vivo PS delivery cancer treatment	Double light wavelength for PTT and PDT, PS coated GNRs, nontargeted	[6,20,21]
Gold nanocages	45 nm edge length, 5 nm wall thickness	64%	0.4 W/cm^2^, 808 nm	[9]	PTT	In vitro cells eradication	Specific targeted, NIR wavelength	[22]
						In vivo cancer treatment	PEG coated nanocage specific targeted, NIR wavelength	[23]
					PDT	In vitro and in vivo PS delivery, cancer treatment	Double light wavelength for PTT and PDT, PS coated nanocages, nontargeted	[5,24]
Gold sphere	20 nm	97–103%	0.28 W, CW laser, 532 nm	[25]	PTT	In vitro cell eradication	Specific targeted, NIR fs wavelength	[26]
						In vitro cell eradication	Targeted cells with two specific antibodies to form nanocluster Visible and NIR wavelength	[27,28]
						In vitro cell eradication	Specific targeted, visible wavelength	[29,30,31]
Gold nanoshell	50 nm	59%	815 nm	[13]	PTT	In vivo cancer treatment	PEG coated, nontargeted, NIR wavelength	[32,33,34,35,36]
	145 nm	25%	2 W/cm^2^, 808 nm	[15]		In vitro cell eradication	PEG coated, specific targeted, NIR wavelength	[37,38]
	154 nm	30%	815 nm	[13]		In vitro core of solid tumors treatment	Au-laden monocytes/macrophages, NIR wavelength	[39]
	152 nm	39%	CW laser, 2 W/cm^2^, 810 nm	[40]	PDT	In vitro, PS delivery, cells eradication	A two-photon femtosecond pulsed laser for both PTT and PDT, PS coated GNRs, nontargeted	[41]
						In vitro cells eradication	Double light wavelength for PTT and PDT, specific targeted	[7,42,43,44]
Gold nanoflower	145 × 123 × 10 nm	74%	1 W/cm^2^, 808 nm	[45]	PTT	In vitro and in vivo cancer treatment	Nontargeted, NIR wavelength	[46,47]
Gold nanoring	400 nm	56%	CW laser, 0.1 W/mm^2^, 700–900 nm	[48]	PTT	In vitro cell eradication	Specific targeted, NIR wavelength	[49]
